# Australian trans and gender diverse people’s understandings of menopause: beyond cisgenderism, heterosexism, and binary-based gender in the current ‘menopause moment’

**DOI:** 10.1093/heapro/daag132

**Published:** 2026-09-01

**Authors:** Kerryn Drysdale, Yeşim Karasu, Lucy Watson

**Affiliations:** Centre for Social Research in Health, UNSW Sydney, Kensington, NSW 2052, Australia; Australian Human Rights Institute, UNSW Sydney, Kensington, NSW 2052, Australia; ACON, Sydney, 414 Elizabeth Street, Surry Hills, NSW 2010, Australia; ACON, Sydney, 414 Elizabeth Street, Surry Hills, NSW 2010, Australia

**Keywords:** gender affirming healthcare, gender affirming hormonal therapies (GAHT), menopause hormonal therapies (MHT), health equity, trans men, non-binary people, qualitative research

## Abstract

In many high-income countries, including Australia, intensified political, social, and commercial visibility has been widely described as contributing to a contemporary ‘menopause moment’. This moment is propelled by the convergence of varied social, political, and economic drivers, but it is also unfolding within a context shaped by historically gendered assumptions about who experiences menopause and how it is experienced. While the current ‘menopause moment’ has raised awareness across a range of domains, there is also the risk of the amplification and normalisation of a particular type of person who experiences a particular type of menopause. Drawing on three focus groups conducted with 17 trans men and non-binary people with ovaries, this article attempts to intervene in the universalizing tendencies on which prevailing (and pervasive) discourses on menopause circulate. By paying close attention to how transgender and other gender diverse people defined, applied, and potentially resisted such discourses, we reveal how understandings of menopause among trans people were mediated both by their and others’ gender, and by the different contexts in which people experience hormonal change, including their experiences of gender affirmation. The contemporary discourses around menopause may be at a tipping point where paradoxically, in the wake of the heightened visibility of menopause, there is a risk of the increasing narrowness of the conversation. Now is the time to move menopause beyond cisgenderism, heterosexism, and binary-based gender so that trans people can be included in this contemporary ‘menopause moment’ as a matter of equitable health promotion.

Contribution to Health Promotion

- The contemporary ‘menopause moment’ is shaped by historically gendered assumptions about menopause.
- Trans participants’ understanding of menopause was mediated both by their gender and the different contexts in which they experience hormonal change.
- In the wake of the heightened visibility of menopause, the current ‘menopause moment’ risks narrowing the conversation and normalizing only one type of menopause.
- This menopause moment needs to go beyond cisgenderism, heterosexism, and binary-based gender assumptions so that trans people’s experiences are considered as a matter of health promotion equity.

In many high-income countries, including Australia, intensified political, social, and commercial visibility has been widely described as contributing to a contemporary ‘menopause moment’ (Orgad and Rottenberg 2024a, Mead 2025, Santoro 2025). Such circulating discourses often rest on competing and conflicting framings that position menopause as both ‘exceptionalised’ (by virtue of potentially debilitating symptomatology and lifecourse interruption) and ‘unexceptional’ (as it is seen as a process of biological aging and an inevitable aspect of being a person with ovaries) (de Salis *et al*. 2018, Thomas *et al*. 2024). However, this ‘menopause moment’ is also shaped by historically gendered assumptions about who experiences menopause and how those experiences should be understood and anticipated—assumptions that continue to centre cisgender women at midlife when menopause signals an end to their reproductive capacity (Stotland 2002, Riach and Jack 2021, Whiley *et al*. 2023, Westwood 2024, 2025, Thomas *et al*. 2025, Wood *et al*. 2025, Drysdale *et al*. 2026b).

Menopause is clinically defined in relation to the date of the final menstrual period and consequent cessation of ovarian function (Stuenkel *et al*. 2015). As a retrospective diagnosis, menopause refers to a period 12 months since an individual**’**s last menses (typically occurring between the ages 45 and 55), while perimenopause, the period when symptoms can begin, can stretch for a long period of time before menopause can be definitively identified (World Health Organization 2024). Further delineations, such as premature (before the age of 40) or early (between the age of 40 and 45) menopause, caused by premature ovarian insufficiency, chromosomal abnormalities, and other health conditions, also contribute to lay understandings of menopause as an event at (or at least around) midlife. Comparably, the range of induced experiences of menopause as a result of biomedical interventions are not as visible in this ‘menopause moment’ to the same degree.

Some transgender and gender diverse (hereafter trans) people with ovaries will experience menopause associated with biological aging. Yet, menopause-like experiences among trans people also occur across a wider range of physiological pathways than those typically described in cisgender populations, including those who experience menopause as part of a suite of interventions related to gender affirming hormonal therapies (GAHT) (Cizek *et al*. 2017, Windt *et al*. 2024 ). Experiences include surgical menopause following oophorectomy, pharmacologically induced menopause (e.g. GnRH analogues), and fluctuations associated with initiation, cessation, or interruption of GAHT—all of which precipitate abrupt oestrogen fluctuation post-puberty that produces menopause-like symptoms outside conventional age or lifecourse expectations (Coleman *et al*. 2022).

Recent literature also emphasises how menopausal experiences are shaped by other factors, such as socioeconomic status, race and ethnicity, geography, disability, and health literacy and associated (in)vulnerability to commercial pressures (Hoga *et al*. 2015, Ilankoon *et al*. 2021, Harlow *et al*. 2022, Li *et al*. 2023, Lycke and Brorsson 2023, Matina *et al*. 2024, Corus *et al*. 2025, Schneider Dallolio *et al*. 2025, Takhar *et al*. 2025a, 2025b, Thomas *et al*. 2025). Indeed, global literature demonstrates how different cultural contexts produce different experiences of menopause (Mackey *et al*. 2014, Amini and McCormack 2019, Ussher *et al*. 2019, Zou *et al*. 2021, Harlow *et al*. 2022, Li *et al*. 2023, Lycke and Brorsson 2023, Matina *et al*. 2024). Understanding the impacts of these intersecting factors is important as they affect how an individual anticipates their experience of menopause (Nappi *et al*. 2021, Harlow *et al*. 2022). Accordingly, there is no universal experience of menopause. Moreover, Hickey and colleagues show that (over)medicalisation of menopause risks collapsing the wide range of experiences into a narrowly defined disease, which, in turn, emphasises (and potentially catastrophises) its negatively embodied experience (Hickey *et al*. 2022, Thomas *et al*. 2025). At the same time, the current menopause moment—fuelled by media, popular, and commercial discourses—tends towards universalising all experiences into those (only) requiring a common response (Hickey *et al*. 2022, Davis and Magraith 2023, Thomas *et al*. 2024).

 

### The current menopause moment in Australia and beyond

Concomitant with the rise in its visibility, attention is increasingly paid to menopauses’ various effects and impacts, which has in part led to increased dissemination of information on menopausal symptomatology and its management. Recent analyses have shown that such heightened visibility intensifies pharmaceutical, wellness, and productivity-oriented discourses ( Rowson *et al*. 2023, Orgad and Rottenberg 2024a, 2024b).

That menopause, as a product of biological aging, can potentially be delayed or reversed by pharmaceutical intervention is often reinforced in health consumer information (Lyons and Griffin 2003, Amini and McCormack 2019, Krajewski 2019, Rowson *et al*. 2023, May 2024, Randle *et al*. 2024, McCartan 2025, Schneider Dallolio *et al*. 2025, Takhar *et al*. 2025a, 2025b). Not surprisingly, menopause has also captured the attention of the commercial wellness industry seeking to capitalise on menopausal treatment regimens. These commercial drivers adopt neoliberal models of responsible health citizenry, which drive market demand for its products - effective or otherwise (Padamsee 2011, Rowson *et al*. 2023, Corus *et al*. 2025, Schneider Dallolio *et al*. 2025). Along with considerable potential for misinformation around menopause (Herbert *et al*. 2020, Van Poucke 2025), commercial interest is making menopause somewhat ubiquitous, with algorithmically determined advertisement targeting women at midlife. This could have a paradoxical effect of unduly influencing purchase decisions at the same time as expecting people to function as market-savvy consumers (Corus *et al*. 2025, Onculer and Onculer Yayalar 2025, Schneider Dallolio *et al*. 2025, Takhar *et al*. 2025b, Thomas *et al*. 2025).

Conversely, health policy analysts and economists draw attention to menopause as not only as an individual health event, but as an issue linked to labour participation and economic productivity (Vicki Kafanelis *et al*. 2009, Riach and Jack 2021, Riach and Rees 2022, Whiley *et al*. 2023, Thomas *et al*. 2024, Goswami and Cherrier 2025, McCartan 2025, Taylor *et al*. 2025, Walker-Bone and Davis 2025, Wood *et al*. 2025). In some high-income countries, especially, discourses on the economic impacts of menopause identify the collective toll of the decline in individual productivity, of which responses pivot on removing or reducing barriers in the workplace (Riach and Jack 2021, Rowson *et al*. 2023, Whiley *et al*. 2023, Vincent *et al*. 2024, Taylor *et al*. 2025, Walker-Bone and Davis 2025, Wood *et al*. 2025). For example, the Australian Senate Community Affairs References Committee’s 2024 inquiry into menopause and perimenopause debated whether paid reproductive leave should be mandated, which the federal government noted in their response to its recommendation (Community Affairs References Committee 2024, Australian Government 2025). The Australian Public Service Commission has issued national guidance for Australian Government agencies to support employees experiencing symptoms of menopause in the workplace (Australian Public Service Commission 2025). While Australian unions are campaigning for reproductive health leave, including paid leave for menopause for all workplaces, public sector agreements now enshrine these entitlements in standard awards in some state jurisdictions (Molloy 2025).

Menopause can also mark the moment in the lifecourse where systemic inequities can be activated, including disparities in diagnosis and treatment access. Menopause is thus increasingly viewed through a health equity lens. In 2023, Davis and Magraith reported a startling figure: over 85% of Australians with moderate to severe negative symptomatology associated with menopause were not receiving effective, approved, and evidence-based therapies (Davis and Magraith 2023, see also Worsley *et al*. 2016). Menopause Societies (specialist authorities for menopause and post-menstrual health) have now been established in various international jurisdictions, such as Australia, to advocate and amplify calls for improved clinical literacy and community awareness ‘for women through midlife, menopause, and beyond’ (Australasian Menopause Society 2025). In efforts towards increasing access to quality healthcare related to menopause, on 1 July 2025, the Australian government introduced new Medicare Benefits Schedule item numbers for menopause-related health assessments to facilitate subsidised access to menopausal health provision in general practice (Australian Government 2025).

Yet, all of these discourses that identify the individual and collective impacts of menopause often rest on assumptions about what menopause is and who it will impact—and these assumptions can even carry over to research that evidences these impacts (see, e.g. Drysdale *et al*. 2026a). This article is guided by concerns that trans people may be left out of the current menopause moment if we continue to use the term menopause uncritically, which often operates on and consolidates axes of aging, gender, and sexuality. In doing so, there is a risk conflating the diversity of experience into a singular notion of menopause that will continue to exclude outlier or nonnormative experiences. This conflation does a disservice to cisgender heterosexual women who may have experiences that do not conform to dominant assumptions about menopause (see, e.g. Boughton and Halliday 2008), let alone how such assumptions can serve to entrench ongoing and systemic inequities in healthcare among trans people (see, e.g. Drysdale *et al*. 2026b).

The contemporary discourses around menopause may be at a tipping point. This study was designed to address the following questions:

1. What are the experiences and/or expectations of perimenopause and menopause among trans people?
2. What changes are required to menopause resources to better align with these experiences and/or expectations?

In this article, we ask: can the ‘menopause moment’ move beyond cisgenderism, heterosexism, and binary-based gender?

## Methods

This study had been designed to explore the perspectives and experiences of trans people who will or have experienced perimenopause, menopause, and/or other acute hormonal fluctuations associated with oestrogen cessation and/or ovulation cessation. A previous article from this study was focused on participants’ views on menopause-related resources and information seeking practices of trans people (Drysdale *et al*. 2026b).

Qualitative research of this nature is useful when the goal of research is exploratory and approaches to analysis need to be grounded in participants’ perspectives (Braun and Clarke 2006). Focus groups were selected as they are a highly effective method for gaining information about the variety of experiences and opinions about health issues and enabling cross-fertilisation of ideas within a cohort, allowing for synergistic responses and flow of ideas (Kitzinger 1994, Wilkinson 1998). In addition, for research involving trans people, focus groups are useful in alleviating risks associated with disclosing personal information or sensitive medical histories, as participants are encouraged to couch responses in terms of hypothesis and conjecture. All members of the research team were recorded female at birth and have various lived experience of menopause and/or intersections with gender affirming healthcare. Collectively, we were interested in menopause given our own departure from many of the assumptions of menopause, and so were highly attuned to exploring nonnormative experiences, such as this study represented. This facilitated the level of reflexivity required of exploratory qualitative research.

Data were collected using purposeful sampling, with recruitment taking place through closed email lists and social media maintained by ACON, Australia’s largest LGBTQA+ community health organisation. Recruitment and data collection processes were further supported by the involvement of dedicated ACON staff who work on trans health equity matters. The researchers included a positionality statement on all information for participants, noting the gender, sexuality, pronouns, and expertise of each researcher. The research purposefully used expansive definitions of menopause and eligibility criteria in the recruitment materials to invite as much diversity of lived experiences as possible, noting that experiences and/or expectation may differ from with the dominant narratives attributed to cisgender women. Participants were directed to an online form to express their interest in participating, with implied consent to collect demographic information (though completing all questions was not mandatory and did not bar people from participating in focus groups). Informed consent was collected through a signed copy of the consent form prior to commencement of the focus group via email, or by undertaking a verbal consent process before focus groups commenced.

Focus groups were conducted on video conferencing software Zoom (one focus group was conducted online and in person at ACON’s offices simultaneously), lasting between 1 h and 22 min and 1 h and 24 min after consent processes, administration and housekeeping, and introductions were conducted. Focus group discussion was facilitated by both first and second authors and was guided by an interview schedule that posed questions related to: current understandings of menopause and perimenopause; perspectives on menopause-related resources and information available; and current or anticipated menopause-related support and service needs and access. All focus group participants received an AUD $80 gift voucher for reimbursement of their time and in recognition of their lived experience expertise.

All data generated were analysed using reflexive thematic analysis (Braun and Clarke 2021, Braun *et al*. 2022), and coded both deductively in response to the research questions and inductively to identify other relevant themes—using a hybrid approach to thematic analysis to ensure rigor and triangulation (Fereday and Muir-Cochrane 2006). Specifically, we employed Braun et al. (2022) six phased process for reflexive thematic analysis: familiarisation with data through (re)reading each transcription in full; initial coding into categories; identifying patterns evident through coding; generating cross cutting themes; refining themes; and writing up the results. Throughout, we critically reflected on how our own lived experiences shaped the research design, descriptions of menopause, and decisions around participant eligibility. Thus, we consider our analytic process to be inclusive of both experientially (i.e. facilitating participant-led sense making processes) and critically oriented analysis (i.e. interrogating how participants and researchers interpreted their own and others’ experiences; see Braun *et al*. 2022). For this article, we present data predominately from coding nodes related to definitions, categorisations, and direct experiences of menopause. Ethical approval to undertake this research was received from the UNSW Human Research Ethics Committee (iRECS7219) and the ACON Research Ethics Review Committee (D202408).

## Results

Seventeen participants took part in three focus groups conducted in October 2024. Purposeful sampling resulted in a cohort with representation of diverse demographic characteristics, including age, gender, sexuality, geographical location, and country of origin (see Table 1). All participants were recorded female at birth, but equal numbers of trans men and non-binary participants showed relevance to both populations. The higher percentage of younger people aged 18–29 (35% of the sample) reflected younger trans people’s interest in and expectations of menopause that aligned with how they perceived their experiences before midlife. The next greatest percentage were among those thought to experience perimenopause at ages between 40 and 49 (24% of the sample), which highlighted for some participants their immediate and direct experiences that align with normative assumptions around cisgender women’s experiences at midlife. Common among this cohort were experiences of surgically induced menopause (i.e. oophorectomies carried out with hysterectomies) and the sudden cessation of menstruation through the advent of gender affirming hormonal therapies (i.e. commencing testosterone). Almost a third of the participants were born overseas, however, all were from high-income countries comparable to Australia (United Kingdom, United States of America, and Aotearoa New Zealand). While participants provided their preferred terms for their gender, the basic demographic information accompanying each quote is simplified to man or non-binary (as the majority-preferred terminology) to avoid re-identification of participants.

**Table 1:** Participant demographic data.

Characteristics	*n*	%
Age		
18–29	6	35
30–39	2	12
40–49	4	24
50–59	2	12
60–69	1	6
Prefer not to answer	2	12
Gender^a^		
Man	6	35
Non-binary	6	35
Agender	1	6
Another term	4	24
Sexuality		
Queer	11	64
Bisexual	3	18
Pansexual (or variations of)	3	18
Country of birth		
Australia	9	53
Other (UK, NZ, USA)	5	29
Prefer not to answer	3	18
Location of residence		
Urban	12	71
Regional	2	12
Rural	1	5
Prefer not to answer	2	12
Total	17	100

^a^All participants were recorded female at birth.

In this article, we look to how menopause is understood and made meaningful among trans participants. This is evident in three thematic categories related to menopause: definition, application and resistance.

### Defining menopause through clinical and vernacular frameworks

When asked to define menopause, participants frequently drew on definitions closely aligned with clinically oriented diagnoses:

[Menopause is a] medical kind of statement, that it's after the cessation [of] your last period […]. And they say that completed menopause [is when] you've had no periods for a year. (Man, aged 30–39)

From my understanding, menopause is 12 months after your last menstruation, basically when the body ages out of reproducing, like producing eggs and things like that. (Non-binary, aged 40–49)

These two quotes were representative of participants’ familiarity with conventional biomedical framings: menopause as a moment that can only be identified retrospectively and as a chronological transition linked to reduced ovarian function. Perimenopause was similarly defined by participants according to such clinical frameworks—as the period from when changes to menstrual cycles are first observed to one year after the final menstrual period. Phrasing such as ‘medical kind of statement’, ‘they say’, and ‘from my understanding’ indicated that participants were comfortable drawing on these clinically derived narratives, suggesting recourse to standard definitions.

Under these diagnostic frameworks, however, participants queried whether menopause is applicable to their own experiences over the lifecourse, even when they were at an age conventionally associated with menopause:

I have had a hysterectomy recently […]. And so, a lot of these things don't apply to me. Like, I don't have a cervix anymore. I don't have ovaries anymore. (Non-binary, aged 50–59)

As such, participants did not necessarily disavow menopause *per se* but relied on precise clinically derived details to properly determine the criteria for definition. At the same time, many participants identified ambiguity over the definition of menopause when identifying how the terms perimenopause and menopause were used in everyday vernacular. Specifically, participants recognised that these two clinical terms were often conflated, or that menopause was often used as shorthand to refer to the longer transitional period of perimenopause:

But everyone kind of describes menopause [to be] what is actually perimenopause, which is when your hormones are shifting as the body is going through that hormonal change, working up to having no periods for a year. (Man, aged 30–39)

I didn't come across the word [menopause] compared to perimenopause, but I understand it to be the fluctuation [of] perimenopause. But I don't really think of them as being separate. (Man, aged 18–29)

Confounding factors therefore emerged between consensus on the definition of menopause. This was most evident between clinical precision and vernacular conflation, with ‘menopause’ frequently functioning as shorthand for a longer period of hormonal fluctuation rather than referring to a discrete post-menopausal state. This tension was more pronounced when the conversation moved to the implications of these potentially ambiguous or conflated meanings derived from either clinical and/or vernacular descriptions. This tension arose in three ways.

First, participants broadly resisted the notion that menopause can only ever be the product of biological aging. Instead, menopause was conceptualised as a broader process of hormonal fluctuation or oestrogen cessation that could occur through multiple pathways:

Yet, it's something that every AFAB [assigned female at birth] human is going to go through at some point, be it earlier because you go on testosterone, or later because the aging process. (Non-binary, aged 60–69)

Second, participants also recognised that menopause was dominantly associated with a particular moment in the lifecourse, attributable to cisgender women at midlife:

I strongly believe that we hold the word menopause to a very direct age group, and I think just reading general things online about it, everyone’s like, ‘yeah, happens right after you're 40’. (Non-binary, aged 18–29)

But as this same participant continued to say, by reframing menopause in terms of its physiological effects, rather than as a universal lifecourse event, participants were therefore more likely to attribute their own physiological symptoms to an earlier stage in life:

But myself, as soon as I started testosterone. I'm around 18 now, that is when I started going through it. So, I think we need to open up the possibility that a lot of younger people are going through that. (Non-binary, aged 18–29)

Third, participants were highly opposed to any distinction between ‘natural’ and biomedically induced menopause:

For me, the experience of menopause is just a natural process I've been through as part of my gender affirming journey. (Man, age not provided)

Participants resisted distinctions between ‘natural’ menopause and hormonally mediated bodily transition because participants perceived that biomedical interventions brought on or accelerated menopause before midlife. Instead, menopause-like experiences were frequently understood as part of a continuum of hormonal change that included gender-affirming interventions. In the same vein, other participants challenged the notion that any menopausal experiences can ever be considered ‘natural’. Instead, menopause was understood as a physiological process requiring some form of diagnosis (and potentially treatment), which could occur through multiple hormonal pathways:

I don't think it's like really a natural thing. I think there's so many things because [menopause] is caused by other things as well. Why should that be natural? (Non-binary, aged 50–59)

As this participant continued, the basis of querying the existence of ‘natural menopause’ was the implication that some forms of menopausal experiences are ‘unnatural’. As they had already commenced menopausal hormonal therapies—‘the fact that I would like hormones to replace my previous hormones’—menopause was seen by this participant as just one pathway of a greater diversity of potential biomedical interventions, regardless of age.

Menopause therefore emerged through the focus group discussions not as a singular or universal biomedical event, but as a flexible and contested umbrella term through which participants could interpret diverse forms of hormonal change. Such umbrella terms, compared to clinically precise terminology, encapsulated a diversity of meanings when viewed through the lens of symptomatology and treatment. As such, there was consensus that trans experiences should not be ‘othered’ through definitions of menopause attributed to midlife experiences, reproductive capacity, or ‘natural’ aging. Doing so can result in stigma, discrimination, and exclusion if menopause is only ever spoken about in terms of natural versus unnatural on which many lay interpretations rest:

I feel like it further stigmatises [me] and other people who don't experience menopause in the textbook way. (Non-binary, aged 18–29)

### Applying the symptoms of menopause through a gender affirmation lens

For many participants, their own menopausal-like symptoms, perceived as physiological effects, were understood as part of the same continuum of hormonal fluctuation that they experience through other biomedical interventions. That is, participants recognised that their own physiological experiences, which occurred for them after undergoing surgical interventions (oophorectomies) or undertaking gender-affirming hormonal therapies (testosterone), were similarly aligned with menopause symptomology:

Menopause is also about what can happen after something like a hysterectomy, where you don't know if you're still bleeding or not, but your body has stopped doing that for other reasons. So, whether that's surgical reasons, whether that's some medical treatments as well, [those reasons] can cease [stop] the body producing those hormones. I sort of reached estrogen cessation, but mainly it was the advent of going on T [testosterone]. And there's the people I speak to [who] tend to understand that [gender-affirming hormonal therapeutic intervention] also means that there's sort of a pseudo menopausal effect. (Man, aged 18–29)

Conversely, when solely relying on clinical definitions, biomedical interventions that produced similar symptoms meant that it was difficult for participants to distinguish the reason for their sudden onset of menopausal symptoms. In these cases, participants tended to revert to vernacular uses of menopause as an umbrella term as it could accommodate the way that they expressed physiological and psychological ambiguity:

It's really hard to tell because I haven't bled for, you know, over a year and a half now. So yeah, and even before that, I was skipping as many periods as I could due to other medical stuff. So, yeah, it is weird to try and pinpoint that. (Non-binary, aged 40–49)

This comment also raises the possibility of menopause to be brought on by ‘other medical stuff’ outside of gender affirmation interventions. Participants acknowledged that this could be, for instance, where menopause may be induced through treatments for hormonally sensitive cancers or in response to menstruation-related considerations for people living with other chronic health conditions, such as polyendocrine metabolic ovary syndrome (PMOS) or endometriosis/adenomyosis. At the same time, the specificity of the effects of menopause—that is, its physiological symptoms—was seen a more precise way of describing menopause rather than relying on broader meanings and associations seen through the cessation of menstruation. Participants whose menstruation had already been altered through gender-affirming hormonal therapies or other interventions described difficulty locating themselves within existing definitions of menopause if it is only ever aligned or concomitant with menstruation. This is because participants identified that, used in this way, menopause in both clinical and vernacular uses often served as a proxy for menstruation cessation.

Much more relevant to participants was how menopause could be potentially interpreted through the lens of gender affirmation, particularly where hormonal fluctuation, menstrual cessation, and bodily changes intersected with experiences of gender-affirming care. For many participants, menopausal experiences were integral to understanding the impact of their own gender-affirming interventions. This perception is evident in one participant’s equation of menopause as part of the process of affirming their gender:

[Perimenopause and menopause] are largely part of the same experience, [and] it was an accelerated experience made because of testosterone. […] I only really started thinking about it because I started having atrophy symptoms on testosterone recently and so started being treated [with] topical estrogen. And so that was when I started to conceptualise that it [menopause] is a way of thinking about a process that my body has gone through, and it's an umbrella term that a lot of atrophy and topical estrogen treatment is kind of filed under. (Non-binary, aged 18–29)

This ambiguity complicated conventional biomedical definitions of menopause that rely on menstrual cessation as a retrospective diagnostic marker. Instead, many participants interpreted these experiences through clinically oriented framings of hormonal fluctuation and oestrogen withdrawal. Importantly, however, was the way that participants often described their experiences of hormonal fluctuation and oestrogen withdrawal positively, particularly where menstrual cessation affirmed their gender:

I can't wait for my periods to stop. You know what I mean? I don't I can't wait for that all to be over. So, in my mind, it [menopause] is a really positive thing. (Non-binary, aged 40–49)

For such participants, bodily changes associated with menopause or hormonal fluctuation could be experienced as positive when interpreted through the framework of gender affirmation.

However, these experiences were not universal. Some participants distinguished sharply between gender-affirming hormonal care and menopausal-related care, particularly where menopausal interventions did not align with their gender:

I find it I completely distinguishable between taking testosterone as being part of my gender affirming care, and I consider that entirely separate to the topical estrogen that I'm taking as being part of the additional care that I'm receiving, because it doesn't feel gender affirming to me. (Man, aged 18–29)

This distinction demonstrates that participants did not uniformly adopt menopause as an explanatory framework that defined what their bodies were going through. Rather, menopause was only ever applicable through participants’ perspectives on whether a particular intervention that resulted in, and their embodied experiences of symptoms of, menopause aligned with participants’ understandings of gender affirmation. For those participants who firmly perceive menopause, as either a clinical term or in its vernacular use, as an experience associated with biological aging and reproductive decline, they were forced to distinguish between interventions designed to affirm their gender from interventions designed to alleviate the negative symptoms of menopause. This created an impossible binary, one in which one is received positively while the other is pathologised. Such distinctions then work to allocate one form of hormonal therapy to gender affirming treatments, while others are relegated to menopausal treatments; in effect, separating the function of treatments, regardless of their underlying similarities to treat particular physiological effects. This siloing of the treatment regimens was also a source of frustration in ways that trouble recourse to easy distinctions: all participants expressed frustration that healthcare systems and resources frequently separated menopause care from gender-affirming care, thereby reinforcing experiences of marginalisation:

I think that every single GP who deals with me should be able to deal with the fact that when I want to balance my hormones that comes into my gender identity and that I can do what the fuck I want. They should stop telling me it's not HRT [hormone replacement therapy], it's MHT [menopause hormonal therapy]. All of them should do that. (Non-binary, aged 50–59)

In this sense, participants advocated for more integrated and affirming approaches to menopause care that recognised hormonal fluctuation and menopause-like symptomology as potentially intersecting with gender-affirming healthcare. This challenges both clinical and lay divisions of two healthcare pathways as separate in ways that hold that trans experiences of similar experiences of menopause are either nonsensical, peripheral, or exceptional.

### Resisting dominant social scripting of menopause

Alongside applying menopause through frameworks of gender affirmation, participants resisted dominant social scripts that positioned menopause as a universally negative experience. In this latter sense, participants frequently described mainstream menopause discourse as not relevant to their own experiences. For many participants, the term menopause itself carried social meanings associated with aging and reproductive failure that did not resonate with them:

I would prefer not to call it menopause. It makes me really shitty. Like it is a hormonal deficit in the same way that a thyroid hormone deficit can occur as you get older? And yes, it happens to half of the people in the world, which is fine, but defining it as anything as anything other than a hormone change really bothers me. (Non-binary, aged 50–59)

The basis of this resistance lay in the medical pathologisation of menopause:

I think that the whole concept of menopause is so pathologised. I mean the way it's called symptoms. So, I can't think of a better word, but it implies that it's an ailment. (Non-binary, aged 60–69)

This quote, and the one below, challenges dominant heteronormative framings that reduce menopause to a byproduct of reproductive decline, couched in terms of hormonal deficiency. Participants perceived this framing to be reductive and a patriarchal-induced interpretation of women’s social, political and economic roles:

I'm extremely shitty with the idea that menopause is what marks the end of a woman's reproductive life. Like, no, it's a hormonal fluctuation. (Non-binary, aged 50–59)

As this participant made clear, clinical definitions are based on changes to ovarian function but that this function should not stand in as proxy for a decline in reproductive capacity. Indeed, for some trans people, reducing or ceasing testosterone-based hormonal therapies can reintroduce ovarian function and potentially reverse their menopausal experiences, which disrupts any familiar or singular narrative of menopause as disrupting womanhood, fertility, and femininity. Participants astutely identified culturally oriented definitions of, or discourses around, menopause, especially where those applications were correlated to women’s reduced social, political, and economic roles in society where any cultural benefit that can be derived was tied to the value of reproduction.

As such, participants were already drawing on social and cultural scripts that aligned with—yet exceeded—clinical definitions of menopause, refusing to constrain any and all experiences of menopause to those predominately associated with heterosexual reproduction. Rather, participants reframe menopause as a physiological phenomenon that is not inherently tied to womanhood or fertility. These accounts suggest that participants resisted dominant menopause discourse not necessarily by rejecting bodily change caused by ovarian hormonal shifts, but by challenging the social meanings attached to menopause. Notably, participants also challenged deficit-oriented framings of menopausal symptoms, preferring language that conceptualised bodily experiences as changes over time:

They [symptoms] may not apply. They indicate presence and absence versus change, which is really important for people who have had other experiences around these. (Man, aged 18–29)

Instead, some participants preferred the language of ‘hormonal fluctuation’ because it was perceived to be more neutral and less socially ladened:

It's about the hormonal fluctuations. […] That's a really important distinction. It's not a decline. (Non-binary, aged 50–59)

Referring to menopause as hormonal fluctuation was seen to be a safer, more accurate conversation to have for trans people.

Participants therefore resisted assumptions that menopause was a singular or universal experience. Instead, they emphasised diversity and individual variability in symptomology and embodiment:

I think they need to very much reword what is menopause is and maybe acknowledge that not everyone experiences menopause the same way and has the same symptoms. (Man, aged 18–29)

This reframing was perceived as more reflective of the fluctuating nature of hormonal change in which they similarly saw their own experiences. Accordingly, participants’ accounts suggest that resisting dominant social scripts of menopause involved both rejecting narrow social narratives of menopausal decline and advocating for more inclusive, affirming, and flexible understandings of hormonal fluctuation and embodied change. The way that participants recounted these experiences and perceptions suggest the need to destigmatise menopause—and if warranted, the preference for medical intervention—in line with a patient-centred approach that prioritises agency:

Like, this is a major hormonal change that we're going through, and we can address in our own way. (Man, aged 18–29)

## Discussion

Trans participants in our study revealed how they defined, applied, and potentially resisted a combination of terms, associations, and proxies broadly aligned with ‘menopause’. Generally, participants within focus groups identified a variety of ways that the term was used. Finely grained delineations, often determined by the age associated with the onset of symptoms (premature, early, perimenopause, and post-menopause), were useful for participants in this study to identify biological changes in their bodies, often enabling self-diagnosis that can trigger information seeking practices and encourage engagement with healthcare services (see also Drysdale *et al*. 2026b). Used interchangeably or simultaneously in the same conversation, participants also identified vernacular uses of menopause that function as a broad umbrella term, which allowed participants to refer to their own experiences as menopause. However, opposition was voiced in respect to how menopause was often used as a proxy for a range of other socially mediated discourses. Participants firmly resisted the sociocultural meanings that coupled menopause to reproductive capacity and cisgender womanhood, as they imply socially sanctioned understandings of gender, age, and sexuality (see also similar findings across literature in Brown *et al*. 2015). Attention to sociocultural dimensions of menopause is crucial as research has shown that the expectations associated with menopause in turn influence the embodied experience of it (Lyons and Griffin 2003, Ayers *et al*. 2010, Hunter and Chilcot 2013, Hickey *et al*. 2022).

It is not surprising, then, the term ‘menopause’ may be ambiguously or uneasily received by trans people (see also Toze and Westwood 2025, Drysdale *et al*. 2026a, 2026b). This ambiguity reveals how understandings of menopause among trans people were mediated both by their and others’ gender, and by the different contexts in which people experience hormonal change, including trans people’s experiences of gender affirmation. This could also mean that the cessation of menstruation and the onset of menopause could be experienced as positive among trans people, as they decouple menopause from reproductive function and associated sentiments of decline and dysfunction. While menopause is subject to widespread social discourses that position it as characterised by a decline in reproductive capacity due to hormonal decay, feminist scholarship has long critiqued this tendency to frame menopause primarily through narratives of decline, dysfunction, or reproductive failure (Bell 1987, Martin 2001, Greer 2018). Yet, these findings also support other literature that reveal how trans people may experience uncertainty in recognising and interpreting symptoms as menopausal, particularly when vasomotor or cognitive changes overlap with or are attributed to GAHT or other experiences of (ill)health (Mohamed and Hunter 2019, Cheung *et al*. 2023, Toze and Westwood 2025).

Crucially, these findings from this study contrast with the way that emerging and circulating discourses on menopause tend to rely on a universal experience, which can impact cis and trans people alike. Media, popular, and commercial attention to health issues has a significant impact on health policy and practice, which renders menopause vulnerable to shifting influences that can enable or constrain individual healthcare seeking practices and healthcare service provision. For example, literature has documented the ways in which women and people with ovaries are being targeted by the wellness industrial complex, which, combined with a lack of investment in women’s healthcare more generally, can either leave people vulnerable to misinformation and commercially driven pseudo-medical products while simultaneously demanding their responsibilisation as market-savvy agents and consumers (Corus *et al*. 2025, Onculer and Onculer Yayalar 2025, Schneider Dallolio *et al*. 2025, Takhar *et al*. 2025b, Thomas *et al*. 2025). The (over)medicalization of menopause also can affect patients’ agency and autonomy in determining that best course of action that responds to their own healthcare preferences (Murtagh and Hepworth 2003, Padamsee 2011). Other literature has highlighted the psychosocial and cultural aspects of menopause, extending focus from the ‘silent biological changes’ (Davis and Magraith 2023) to a range of intersectional sociocultural processes and as occurring within highly variable sociopolitical contexts (Flint and Samil 1990, Hunter and Rendall 2007, Hyde *et al*. 2010). In high-income countries, especially, attention is paid to the experiences of menopause in workplaces where it is framed by declines in individual productivity and the economic impacts of people prematurely leaving or taking time away from the workforce (Riach and Jack 2021, Rowson *et al*. 2023, Whiley *et al*. 2023, Vincent *et al*. 2024, Taylor *et al*. 2025, Walker-Bone and Davis 2025, Wood *et al*. 2025). While the current ‘menopause moment’ has raised important awareness across a range of social, political, and economic domains, there is also the risk of the amplification and normalisation of only one type of menopause.

While identifying important distinctions between trans experiences and/or expectation of menopause and the normative assumptions associated with heterosexual cisgender womanhood, more research is needed to carefully disentangle intersectional experiences of ethnicity and culturally mediated experiences, as well as unpacking these experiences from other chronic health conditions which may be distinct from or impact menopause. Equally, there may be important distinctions in understandings of, and service access needs related to, permanent menopause (i.e. no possibility of returning to a state of ovarian function) or temporary menopause (i.e. where menstruation may return if certain biomedical interventions are ceased), not explored here. The absence of clear clinical guidance (Cheung *et al*. 2019, 2023) and limited inclusion of trans bodies in menopause research (Drysdale *et al*. 2026a) contribute to difficulties in distinguishing menopause-related changes from other health, hormonal, or biomedical interventions. Recognising menopause as part of a broader life-course approach to gender-affirming healthcare ensures that emerging menopause discourses do not exclude trans people but may instead contribute to more equitable and inclusive healthcare.

Equity in health promotion is often centred on inclusiveness (Drysdale *et al*. 2024, 2025). This requires a crucial reorientation to be more critical of how we talk about menopause. Understanding menopause as socially and culturally situated, shaped not only by hormonal change but also by broader relations of gender, aging, class, race, sexuality, and embodiment are also critical for trans inclusiveness in understanding menopause (Mohamed and Hunter 2019, Glyde 2022, Cheung *et al*. 2023, Mehta *et al*. 2024, Sobel *et al*. 2024, Toze and Westwood 2025, Westwood 2025, Drysdale *et al*. 2026b). This is because outside of clinical interventions, menopause is also experienced as a sociocultural phenomenon, in which menopause is often the target of other drivers, or can stand in for a range of other proxies. Rather than dismissing these tensions as inevitable via the comparison of clinical versus vernacular uses, we find that menopause can—in its popular evocation—become a meaningful term that invokes a range of social forms, cultural meanings, and hierarchically ordered experiences. This then narrows conversations around menopause to those experiences are most visible, which in turn, becomes those most salient and in need of response.

This menopause moment generates an increasingly lay understanding of ‘natural’ (World Health Organization 2024) menopause; that is, the range of menopausal experiences that take place at a result of biological aging. This in turn implies that some experiences are unnatural; for example, those resulting from a range of biomedical interventions. But by making a distinction between natural and unnatural menopause, this necessarily prompts debates over ‘medicalised menopause’ (Hickey *et al*. 2022). This in turn leads to disagreements over whether menopause is being either over- or under-treated, and then indeed, what counts as a symptom of menopause. This divide then invites a separation between menopausal healthcare provision targeting people who may experience hormonal fluctuations at midlife (i.e. cisgender women and people with ovaries) and those who experience the very same experiences of hormonal fluctuation related to biomedical interventions (i.e. trans people and other people who, for a variety of reasons, have undergone biomedically induced menopause). Along with binary-based gender that sees menopause as something that only ‘women’ may experience, current menopausal diagnostic, referral, and treatment pathways may have limited resonance with the complex hormonal changes through which trans people may experience menopause (Toze and Westwood 2025, Westwood 2025, Drysdale *et al*. 2026b). Paradoxically, in the wake of the heightened visibility of menopause there is a risk of the increasing narrowness of the conversation.

## Conclusion

In Australia and other high-income countries, a ‘menopause moment’ has been unfolding amid rising political, social, and commercial visibility that position menopause as a health issue. Yet, this continues to be a moment indexed by aging, gender and sexuality which centre cisgender women at midlife signalling an end to their reproductive capacity. Some trans people with ovaries may experience menopause but this experience often occurs across a range of physiological pathways than those typically described in cisgender populations. While there is no universal experience of menopause, this current menopause moment risk obscuring the diversity of experiences, including those among trans people. Critically interrogating lay understandings of the limited diversity referenced by the shorthand or suppositional function of menopause is essential to prevent this contemporary moment from inadvertently reinforcing exclusion. Now is the time to move menopause beyond cisgenderism, heterosexism and binary-based gender so that trans people can be included in this contemporary ‘menopause moment’.

## Data Availability

The datasets generated and analysed during the current study are not publicly available because they contain potentially identifying information from qualitative transcripts and fieldnotes but are available from the corresponding author on reasonable request.

## References

[daag132-B1] Amini E, McCormack M. Medicalization, menopausal time and narratives of loss: Iranian Muslim women negotiating gender, sexuality and menopause in Tehran and Karaj. Women’s Stud Int Forum 2019;76:102277. 10.1016/j.wsif.2019.102277

[daag132-B2] Australasian Menopause Society . *Australasian Menopause Society*. 2025. https://www.menopause.org.au/

[daag132-B3] Australian Government . *Australian Government Response to the Senate Community Affairs References Committee report: Issues Related to Menopause and Perimenopause*. Australian Government, 2025, 32. https://www.health.gov.au/sites/default/files/2025-02/government-response-to-inquiry-issues-related-to-menopause-and-perimenopause.pdf

[daag132-B4] Australian Public Service Commission . *Circular 2025/02: Supporting Employees Experiencing Symptoms of Perimenopause and Menopause in the Workplace*. 2025, March 28. https://www.apsc.gov.au/resources/circulars-guidance-and-advice/circular-202502-supporting-employees-experiencing-symptoms-perimenopause-and-menopause-workplace

[daag132-B5] Ayers B, Forshaw M, Hunter MS. The impact of attitudes towards the menopause on women’s symptom experience: a systematic review. Maturitas 2010;65:28–36. 10.1016/j.maturitas.2009.10.016 19954900

[daag132-B6] Bell SE . Changing ideas: the medicalization of menopause. Soc Sci Med 1987;24:535–42. 10.1016/0277-9536(87)90343-1 3296222

[daag132-B7] Boughton M, Halliday L. A challenge to the menopause stereotype: young Australian women’s reflections of ‘being diagnosed’ as menopausal. Health Soc Care Community 2008;16:565–72. 10.1111/j.1365-2524.2008.00777.x 18371169

[daag132-B8] Braun V, Clarke V. Using thematic analysis in psychology. Qual Res Psychol 2006;3:77–101. 10.1191/1478088706qp063oa

[daag132-B9] Braun V, Clarke V. One size fits all? What counts as quality practice in (reflexive) thematic analysis? Qual Res Psychol 2021;18:328–52. 10.1080/14780887.2020.1769238

[daag132-B10] Braun V, Clarke V, Hayfield N et al Doing reflexive thematic analysis. In: Bager-Charleson S, McBeath A (eds.), Supporting Research in Counselling and Psychotherapy: Qualitative, Quantitative, and Mixed Methods Research. Cham: Springer International Publishing, 2022, 19–38. 10.1007/978-3-031-13942-0_2.

[daag132-B11] Brown L, Bryant C, Judd FK. Positive well-being during the menopausal transition: a systematic review. Climacteric 2015;18:456–69. 10.3109/13697137.2014.989827 25417543

[daag132-B12] Cheung AS, Nolan BJ, Zwickl S. Transgender health and the impact of aging and menopause. Climacteric 2023;26:256–62. 10.1080/13697137.2023.2176217 37011669

[daag132-B13] Cheung AS, Wynne K, Erasmus J et al Position statement on the hormonal management of adult transgender and gender diverse individuals. Med J Aust 2019;211:127–33. 10.5694/mja2.50259 31271465

[daag132-B14] Cizek S, Nguyen N, Lyon L et al Combined hysterectomy and mastectomy surgery for transgender patients in an integrated health care setting. International Journal of Transgenderism 2017;18:382–388. 10.1080/15532739.2017.1359725

[daag132-B15] Coleman E, Radix AE, Bouman WP et al Standards of care for the health of transgender and gender diverse people, version 8. Int J Transgend Health 2022;23:S1–259. 10.1080/26895269.2022.2100644 36238954 PMC9553112

[daag132-B16] Community Affairs References Committee . *Issues Related to Menopause and Perimenopause*. Commonwealth Parliament. (Australia). 2024. https://www.aph.gov.au/Parliamentary_Business/Committees/Senate/Community_Affairs/Menopause

[daag132-B17] Corus C, Saatcioglu B, Sandikci O. Gendered consumer responsibilization: the constitution of menopausal women as responsible feminine consumer subjects. J Consum Res 2025;52:351–71. 10.1093/jcr/ucae057

[daag132-B18] Davis SR, Magraith K. Advancing menopause care in Australia: barriers and opportunities. Med J Aust 2023;218:500–2. 10.5694/mja2.51981 37330995

[daag132-B19] de Salis I, Owen-Smith A, Donovan JL et al Experiencing menopause in the UK: the interrelated narratives of normality, distress, and transformation. J Women Aging 2018;30:520–40. 10.1080/08952841.2018.1396783 29095126 PMC6191885

[daag132-B20] Drysdale K, Burton-Clark I, Moline K. Reimagining menopause by expanding assumptions shaping research: a scoping review of gender and sexuality diverse people’s experiences and expectations. Int J Transgend Health 2026a;27:162–77. 10.1080/26895269.2024.2447785 41624999 PMC12857664

[daag132-B21] Drysdale K, Creagh NS, Nightingale C et al Inclusive language in health policy—a timely case (study) of cervical screening in Australia. Health Sociol Rev 2024;33:325–41. 10.1080/14461242.2024.2356868 38837699

[daag132-B22] Drysdale K, Creagh NS, Nightingale C et al Beyond words: operationalizing inclusive language in Australian cervical screening health promotion policy. Health Promot Int 2025;40:daaf058. 10.1093/heapro/daaf058 40392562 PMC12090894

[daag132-B23] Drysdale K, Karasu Y, Watson L. Because I felt so alone”: trans and gender diverse people’s needs and preferences for menopausal related information and resources. Int J Transgend Health 2026b:1–14. 10.1080/26895269.2026.261863542619653

[daag132-B24] Fereday J, Muir-Cochrane E. Demonstrating rigor using thematic analysis: a hybrid approach of inductive and deductive coding and theme development. Int J Qual Methods 2006;5:80–92. 10.1177/160940690600500107

[daag132-B25] Flint M, Samil RS. Cultural and subcultural meanings of the menopause. Ann N Y Acad Sci 1990;592:134–92. 10.1111/j.1749-6632.1990.tb30321.x 2197940

[daag132-B26] Glyde T . LGBTQIA+ menopause: room for improvement. The Lancet 2022;400:1578–9. 10.1016/S0140-6736(22)01935-3 36270313

[daag132-B27] Goswami P, Cherrier H. Intimate neoliberal violence and ungrievable meno bodies. J Mark Manag 2025;41:293–311. 10.1080/0267257X.2025.2470830

[daag132-B28] Greer G . The Change: Women, Ageing and the Menopause. London: Bloomsbury Publishing, 2018.

[daag132-B29] Harlow SD, Burnett-Bowie S-AM, Greendale GA et al Disparities in reproductive aging and midlife health between Black and White women: The Study of Women’s Health Across the Nation (SWAN). Women’s Midlife Health 2022;8:3. 10.1186/s40695-022-00073-y 35130984 PMC8822825

[daag132-B30] Herbert D, Bell RJ, Young K et al Australian women’s understanding of menopause and its consequences: a qualitative study. Climacteric 2020;23:622–8. 10.1080/13697137.2020.1791072 32705886

[daag132-B31] Hickey M, Hunter MS, Santoro N et al Normalising menopause. BMJ 2022;377:e069369. 10.1136/bmj-2021-069369 35705221

[daag132-B32] Hoga L, Rodolpho J, Gonçalves B et al Women’s experience of menopause: a systematic review of qualitative evidence. JBI Database Syst Rev Implement Rep 2015;13:250–337. 10.11124/01938924-201513080-00018 26455946

[daag132-B33] Hunter M, Rendall M. Bio-psycho-socio-cultural perspectives on menopause. Best Pract Res Clin Obstet Gynaecol Psychol Issues Obstet Gynaecol 2007;21:261–74. 10.1016/j.bpobgyn.2006.11.001 17161025

[daag132-B34] Hunter MS, Chilcot J. Testing a cognitive model of menopausal hot flushes and night sweats. J Psychosom Res 2013;74:307–12. 10.1016/j.jpsychores.2012.12.005 23497832

[daag132-B35] Hyde A, Nee J, Howlett E et al Menopause narratives: the interplay of women’s embodied experiences with biomedical discourses. Qual Health Res 2010;20:805–15. 10.1177/1049732310363126 20181821

[daag132-B36] Ilankoon I, Samarasinghe K, Elgán C. Menopause is a natural stage of aging: a qualitative study. BMC Women’s Health 2021;21:47. 10.1186/s12905-020-01164-6 33522914 PMC7849153

[daag132-B37] Kitzinger J . The methodology of Focus Groups: the importance of interaction between research participants. Sociol Health Illn 1994;16:103–21. 10.1111/1467-9566.ep11347023

[daag132-B38] Krajewski S . Advertising menopause: you have been framed. Continuum 2019;33:137–48. 10.1080/10304312.2018.1547364

[daag132-B39] Li Q, Gu J, Huang J et al “They see me as mentally ill”: the stigmatization experiences of Chinese menopausal women in the family. BMC Women’s Health 2023;23:185. 10.1186/s12905-023-02350-y 37076835 PMC10116657

[daag132-B40] Lycke A, Brorsson A. Swedish women’s experiences of menopausal transition: a focus group study. Sex Reprod Healthc 2023;35:100807. 10.1016/j.srhc.2022.100807 36528995

[daag132-B41] Lyons AC, Griffin C. Managing menopause: a qualitative analysis of self-help literature for women at midlife. Soc Sci Med 2003;56:1629–42. 10.1016/S0277-9536(02)00165-X 12639580

[daag132-B42] Mackey S, Teo SSH, Dramusic V et al Knowledge, attitudes, and practices associated with menopause: a multi-ethnic, qualitative study in Singapore. Health Care Women Int 2014;35:512–28. 10.1080/07399332.2013.801482 23862640

[daag132-B43] Martin E . The Woman in the Body: A Cultural Analysis of Reproduction. Beacon Press, 2001.

[daag132-B44] Matina SS, Mendenhall E, Cohen E. Women’s experiences of menopause: a qualitative study among women in Soweto, South Africa. Glob Public Health 2024;19:2326013. 10.1080/17441692.2024.2326013 38497205

[daag132-B45] May N . Companies portray menopause as ‘medical problem’ and push women towards ineffective treatments, papers find. *The Guardian*. 2024, March 5. https://www.theguardian.com/australia-news/2024/mar/05/companies-portray-menopause-as-medical-problem-and-push-women-towards-ineffective-treatments-papers-find

[daag132-B46] McCartan A . ‘Holding on’: Postfeminist articulations of menopause in advertising. J Mark Manag 2025;41:343–77. 10.1080/0267257X.2025.2475237

[daag132-B47] Mead R . Menopause is having a moment. *The New Yorker*. 2025, March 3. https://www.newyorker.com/magazine/2025/03/10/menopause-is-having-a-moment

[daag132-B48] Mehta JM, Kanell S, Borowicz CEA et al Transgender patients and gender-affirming hormone therapy through the mid-life. Maturitas 2024;189:108093. 10.1016/j.maturitas.2024.108093 39178607

[daag132-B49] Mohamed S, Hunter MS. Transgender women’s experiences and beliefs about hormone therapy through and beyond mid-age: an exploratory UK study. Int J Transgend 2019;20:98–107. 10.1080/15532739.2018.1493626 32999597 PMC6831003

[daag132-B50] Molloy G . Menopause in the Workplace: The Wellbeing Initiative for Employers of Choice in 2026. *Menopause Friendly Australia*. 2025, October 29. https://menopausefriendly.au/news/menopause-in-the-workplace-the-wellbeing-initiative-for-employers-of-choice-in-2026/

[daag132-B51] Murtagh MJ, Hepworth J. Feminist ethics and menopause: autonomy and decision-making in primary medical care. Soc Sci Med 2003;56:1643–52. 10.1016/S0277-9536(02)00172-7 12639581

[daag132-B52] Nappi RE, Kroll R, Siddiqui E et al Global cross-sectional survey of women with vasomotor symptoms associated with menopause: prevalence and quality of life burden. Menopause 2021;28:875–82. 10.1097/GME.0000000000001793 34033602 PMC8746897

[daag132-B53] Onculer A, Onculer Yayalar E. Menopause on the market: navigating the dualities of care and empowerment. J Mark Manag 2025;41:245–72. 10.1080/0267257X.2025.2460197

[daag132-B54] Orgad S, Rottenberg C. Mediating menopause: feminism, neoliberalism, and biomedicalisation. Fem Theory 2024a;25:338–58. 10.1177/14647001231182030

[daag132-B55] Orgad S, Rottenberg C. The menopause moment: the rising visibility of ‘the change’ in UK news coverage. Eur J Cult Stud 2024b;27:519–39. 10.1177/13675494231159562

[daag132-B56] Padamsee TJ . The pharmaceutical corporation and the ‘good work’ of managing women’s bodies. Soc Sci Med 2011;72:1342–50. 10.1016/j.socscimed.2010.10.034 21435768

[daag132-B57] Randle M, Mintzes B, McCarthy S et al Conflicts of interest in submissions and testimonies to an Australian parliamentary inquiry on menopause. Health Promot Int 2024;39:daae150. 10.1093/heapro/daae150 39569482 PMC11579595

[daag132-B58] Riach K, Jack G. Women’s health in/and work: menopause as an intersectional experience. Int J Environ Res Public Health 2021;18:10793. 10.3390/ijerph182010793 34682537 PMC8536086

[daag132-B59] Riach K, Rees M. Diversity of menopause experience in the workplace: understanding confounding factors. Curr Opin Endocr Metab Res 2022;27:100391. 10.1016/j.coemr.2022.100391

[daag132-B60] Rowson TS, Jaworska S, Gibas I. Hot topic: examining discursive representations of menopause and work in the British media. Gend Work Organ 2023;30:1903–21. 10.1111/gwao.13021

[daag132-B61] Santoro N . Understanding the menopause journey. Climacteric 2025;28:384–8. 10.1080/13697137.2024.2445303 39903209 PMC12319117

[daag132-B62] Schneider Dallolio A, Abdalla CC, Ferraz SB. Bytes of affect: unveiling the emergence of the responsible menopause consumer on social media. J Mark Manag 2025;41:312–42. 10.1080/0267257X.2025.2463496

[daag132-B63] Sobel T, Derakshani D, Vencill JA. Menopause experiences in sexual minority women and non-binary people. Maturitas 2024;185:108007. 10.1016/j.maturitas.2024.108007 38677174

[daag132-B64] Stotland NL . Menopause: social expectations, women’s realities. Arch Women’s Ment Health 2002;5:5–8. 10.1007/s007370200016 12503068

[daag132-B65] Stuenkel CA, Davis SR, Gompel A et al Treatment of symptoms of the menopause: an endocrine society clinical practice guideline. J Clin Endocrinol Metab 2015;100:3975–4011. 10.1210/jc.2015-2236 26444994

[daag132-B66] Takhar J, Muir K, Schneider-Kamp A. “I cannot let this happen to other people”: on menopause advocacy, marketing and consumption with Kate Muir. J Mark Manag 2025a;41:378–87. 10.1080/0267257X.2025.2474833

[daag132-B67] Takhar J, Schneider-Kamp A, Bettany S. All change? The new climacteric market awareness. J Mark Manag 2025b;41:237–44. 10.1080/0267257X.2025.2476834

[daag132-B68] Taylor S, Callahan B, Grant J et al Menopause and work performance: a systematic review of observational studies. Menopause 2025;32:769–78. 10.1097/GME.0000000000002557 40460347

[daag132-B69] Thomas SL, McCarthy S, Wood K et al *“**Menopause is not a dirty word.”* Australian women’s opinions about increased public attention to menopause. SSM Qual Res Health 2025;8:100634. 10.1016/j.ssmqr.2025.100634

[daag132-B70] Thomas SL, Randle M, White SL. (Re)framing menopause: a comprehensive public health approach. Health Promot Int 2024;39:daae052. 10.1093/heapro/daae052 38805675

[daag132-B71] Toze M, Westwood S. Experiences of menopause among non-binary and trans people. Int J Transgend Health 2025;26:447–58. 10.1080/26895269.2024.2389924 40276004 PMC12016236

[daag132-B72] Ussher JM, Hawkey AJ, Perz J. Age of despair’, or ‘when life starts’: migrant and refugee women negotiate constructions of menopause. Cult Health Sex 2019;21:741–56. 10.1080/13691058.2018.1514069 30280959

[daag132-B73] Van Poucke M . Negotiating the maze of menopause misinformation: a comparative analysis of stance in health influencer versus medical professional discourse. Atl J Commun 2025;33:454–76. 10.1080/15456870.2025.2453738

[daag132-B74] Vicki Kafanelis B, Kostanski M, Komesaroff PA et al Being in the script of menopause: mapping the complexities of coping strategies. Qual Health Res 2009;19:30–41. 10.1177/1049732308327352 19029240

[daag132-B75] Vincent AJ, Johnston-Ataata K, Flore J et al A qualitative study of work and early menopause: ‘on-the job’ experiences and career trajectories. Maturitas 2024;182:107920. 10.1016/j.maturitas.2024.107920 38280355

[daag132-B76] Walker-Bone K, Davis S. Menopause, women and the workplace. Climacteric 2025;28:423–30. 10.1080/13697137.2025.2480591 40202406

[daag132-B77] Westwood S . GP services are still heteronormative’: sexual minority cisgender women’s experiences of UK menopause healthcare—health equity implications. Post Reprod Health 2024;30:225–31. 10.1177/20533691241279887 PMC11633061 39251395

[daag132-B78] Westwood S . Lesbian, gay, bisexual, transgender and queer (LGBTQ+) menopause: literature review, knowledge gaps and research agenda. Health 2025;29:468–88. 10.1177/13634593241270923 39115194 PMC12235059

[daag132-B79] Whiley LA, Wright A, Stutterheim SE et al “A part of being a woman, really”: menopause at work as “dirty” femininity. Gend Work Organ 2023;30:897–916. 10.1111/gwao.12946

[daag132-B80] Wilkinson S . Focus groups in feminist research: power, interaction, and the co-construction of meaning. Women’s Stud Int Forum 1998;21:111–25. 10.1016/S0277-5395(97)00080-0

[daag132-B81] Windt IK, Mullens AB, Debattista J et al Developing a gender affirming health response for trans and gender diverse Australians: A qualitative study. International Journal of Transgender Health 2024:1–18. 10.1080/26895269.2024.2374007

[daag132-B82] Wood K, McCarthy S, Pitt H et al *“Hiding symptoms and balancing work, family and relationships”*: Australian women discuss menopause and the midlife collision. Soc Sci Med 2025;387:118681. 10.1016/j.socscimed.2025.118681 41110246

[daag132-B83] World Health Organization . *Menopause*. 2024. https://www.who.int/news-room/fact-sheets/detail/menopause

[daag132-B84] Worsley R, Bell RJ, Gartoulla P et al Low use of effective and safe therapies for moderate to severe menopausal symptoms: a cross-sectional community study of Australian women. Menopause 2016;23:11–7. 10.1097/GME.0000000000000495 26240945

[daag132-B85] Zou P, Waliwitiya T, Luo Y et al Factors influencing healthy menopause among immigrant women: a scoping review. BMC Women’s Health 2021;21:189. 10.1186/s12905-021-01327-z 33957910 PMC8101137

